# Altered brain spontaneous activity in patients with cerebral small vessel disease using the amplitude of low-frequency fluctuation of different frequency bands

**DOI:** 10.3389/fnins.2023.1282496

**Published:** 2023-11-16

**Authors:** Sina Chen, Ruiwang Huang, Mingxian Zhang, Xiaohuang Huang, Shuiqiao Ling, Shuxue Liu, Nan Yang

**Affiliations:** ^1^Zhongshan Hospital of Traditional Chinese Medicine, Zhongshan, Guangdong, China; ^2^Center for Study of Applied Psychology, School of Psychology, South China Normal University, Guangzhou, Guangdong, China

**Keywords:** spontaneous brain activity, amplitude of low-frequency fluctuation, cerebral small vessel disease, mild cognitive impairment, white matter hyperintensity, lacune, cerebral microbleed

## Abstract

**Background:**

Previous studies showed that cerebral small vessel disease (cSVD) is a leading cause of cognitive decline in elderly people and the development of Alzheimer’s disease. Although brain structural changes of cSVD have been documented well, it remains unclear about the properties of brain intrinsic spontaneous activity in patients with cSVD.

**Methods:**

We collected resting-state fMRI (rs-fMRI) and T1-weighted 3D high-resolution brain structural images from 41 cSVD patients and 32 healthy controls (HC). By estimating the amplitude of low-frequency fluctuation (ALFF) under three different frequency bands (typical band: 0.01–0.1 Hz; slow-4: 0.027–0.073 Hz; and slow-5: 0.01–0.027 Hz) in the whole-brain, we analyzed band-specific ALFF differences between the cSVD patients and controls.

**Results:**

The cSVD patients showed uniformly lower ALFF than the healthy controls in the typical and slow-4 bands (*p*_FWE_ < 0.05). In the typical band, cSVD patients showed lower ALFF involving voxels of the fusiform, hippocampus, inferior occipital cortex, middle occipital cortex, insula, inferior frontal cortex, rolandic operculum, and cerebellum compared with the controls. In the slow-4 band, cSVD patients showed lower ALFF involving voxels of the cerebellum, hippocampus, occipital, and fusiform compared with the controls. However, there is no significant between-group difference of ALFF in the slow-5 band. Moreover, we found significant “group × frequency” interactions in the left precuneus.

**Conclusion:**

Our results suggested that brain intrinsic spontaneous activity of cSVD patients was abnormal and showed a frequency-specific characteristic. The ALFF in the slow-4 band may be more sensitive to detecting a malfunction in cSVD patients.

## Highlights

- cSVD patients showed uniformly lower ALFF than controls at typical frequency band and slow-4 band.
- At The typical frequency band (0.01–0.1 Hz), cSVD patients Had lower ALFF In The cerebellum, occipital cortex, and hippocampus compared To healthy controls.

## 1. Introduction

Cerebral small vessel disease (cSVD) refers to an intracranial vascular disease that involves various pathological and neurological processes affecting brain blood vessels (Feng et al., 2021). The disease of cSVD exhibits a high prevalence, which largely exceeds that of large-vessel stroke (Dey et al., 2016). Up to 45% of dementia cases may be brought on by CSVD, which also causes 20% of all strokes worldwide, 25% of which are ischemic strokes (also known as lacunar strokes). It is responsible for roughly 20% of all strokes worldwide and 25% of ischemic strokes (also known as lacunar strokes), of which 20% result in disability for the patient (Pantoni, 2010). Moreover, cSVD is one of main factors leading to vascular cognitive decline and dementia (Cai et al., 2015) and contributing to the pathogenesis of Alzheimer’s disease (AD) (Liu et al., 2018; Kim et al., 2020). Recently, structural magnetic resonance imaging (MRI) techniques have been widely applied to identify brain structural neuroimaging markers associated with cSVD (Chen et al., 2019). Although originating from different pathogenesis, cSVD may commonly exhibit similar structural markers including small subcortical infarcts, vascular lacunes, vascular white-matter hyperintensity, cerebral microbleeds, perivascular space and brain atrophy (Staals et al., 2015; De Guio et al., 2016; Chen et al., 2019). In addition to tremendous endeavors made in the structural field, current studies pertaining to brain functional properties are progressively increasing.

The resting-state functional MRI (rs-fMRI) has been applied to investigate the relationship between cognitive impairment and brain functional activity using the amplitude of low-frequency fluctuation (ALFF) (Zang et al., 2007) in patients with cSVD (Zhou et al., 2020; Feng et al., 2021; Li et al., 2021; Mo et al., 2023). Low-frequency oscillation ranging from 0.01 to 0.1 Hz has been identified as a representative indicator of brain spontaneous activity (Zou et al., 2008; Zuo et al., 2010), and the ALFF, the square root of the power spectrum of the frequency range, is supposed to reflect brain regional spontaneous activity (Zang et al., 2007). Moreover, the regional ALFF changes may function as diagnostic biomarker for cSVD (Feng et al., 2021).

Given the fact that different oscillatory bands usually have different generation mechanisms and different physiological functions (Buzsáki and Draguhn, 2004). The detection of different brain tissues may be more sensitive under a specific frequency spectrum (Zuo et al., 2010; Qi et al., 2020; Sasai et al., 2021). Previous studies (Zuo et al., 2010; Qi et al., 2020) subdivided frequency spectrums of BOLD signals into several frequency bands such as the slow-6 (0.0052–0.01 Hz), slow-5 (0.01–0.027 Hz), slow-4 (0.027–0.073 Hz), slow−3 (0.073–0.198 Hz) and slow-2 (0.198–0.25 Hz) to compare frequency-specific ALFF values in different brain regions. For example, the basal ganglia, thalamus, and precuneus were found to show higher ALFF value in the slow-4 band than that in the slow-5 band (Zuo et al., 2010). Several studies showed a strong association between local brain abnormalities in psychiatric disorders and neural activity in specific frequency bands (slow-4 and slow-5 bands) (Wu et al., 2020; Liao et al., 2021; Ren et al., 2021). Wang et al. (2021) compared the difference in ALFF between AD and aMCI patients under three different frequency bands. They suggest that ALFF in the slow-5 band may be able to help identify severe AD and aMCI. So far, little is known about the functional brain activity of cSVD patients with cognitive impairment in response to different frequency bands. Therefore, we attempted to study the characteristic performance of ALFF values in patients with cognitive impairment at different frequency bands.

This study addressed this problem by examining frequency-dependent neural activity in cSVD patients during the resting state. It is the first study to undertake the spontaneous neural activity of specific frequency bands in cSVD. There are two primary aims of this study: 1. To investigate whether cSVD patients would show abnormal ALFF in regions associated with cognitive function; 2. To ascertain whether these abnormalities would be associated with specific frequency bands. The present study detected the brain regions with ALFF alterations (0.01–0.1 Hz) in cSVD patients contrasting with controls. Based on the specificity of sub-bands, we attempted to reveal the brain regions with abnormal ALFF in slow-4 (0.027–0.073 Hz) and slow-5 (0.01–0.027 Hz), separately.

## 2. Methods

### 2.1. Subjects

We recruited 41 cSVD patients with right-handedness for this study from December 2016 to December 2018 from the inpatient and outpatient of the Neurology Department of Zhongshan TCM Hospital, Guangdong, China. Two experienced neurologists (XH, Huang; SQ, Ling) screened the participants based on the diagnostic criteria. In addition, we also recruited 32 sex-matched healthy participants as the healthy controls (HCs). This study was approved by the Institutional Review Board (IRB) of Zhongshan TCM Hospital (ClinicalTrials.gov identifier: 2016ZSZY-LLK-028). Written informed consent for each patient and the healthy participant was obtained before the study. Table 1 lists the clinical and demographic characteristics of the participants.

**Table 1:** Demography characteristics for the patients with cerebral small vessel disease (cSVD) and the healthy controls (HCs).

	cSVD (*n* = 41)	HCs (*n* = 32)	Value of *p*
Sex (M/F)	23/18	11/21	0.098^a^
Age (years old)	68 *±* 7 (41–80)	42 *±* 15 (25–76)	<0.001^b^
Education level	15/19/5/2	4/6/3/19	<0.001^c^
DSST score	11.48 ± 9.08	N/A	N/A
DST score	10.50 ± 2.56	N/A	N/A
VFT score	17.63 ± 6.77	N/A	N/A
Smoke (Y/N)	23/18	21/11	0.409^b^
Diabetes (Y/N)	15/26	7/25	0.174^b^
Hypertension (Y/N)	28/13	18/14	0.290^b^

Education included a primary school, junior high school, senior high school, university and over. N/A, not applicable. Y/N, Yes or No. Level of education refers to the number of people at each level of education: elementary, middle school, high school, and college. ^a^The value of *p* was obtained by a *χ*^2^-test. ^b^The value of *p* was obtained by a two-sample *t*-test. ^c^The value of p was obtained by a Wilcoxon test.

The cSVD patients were diagnosed according to the Neuroimaging Standards for Research into Small Vessel Disease (Wardlaw et al., 2013). Specifically, the diagnostic standard for imaging of cSVD included: (i) Recent small subcortical infarct: Axial views show an infarct diameter less than 20 mm, which can be larger than 20 mm in the coronal or sagittal view. (ii) Lacunes of presumed vascular origin: round or ovoid in shape, 3-15 mm in diameter, distributed in subcortical regions, filled with the same signals as cerebrospinal fluid (CSF). (iii) white matter hyperintensity (WMH) of presumed vascular origin: abnormal brain white matter (WM) signals, the range of lesions can vary in size, showing a high signal on the T2-weighted or T2-weighted FLAIR images. (iv) Perivascular space: the signal of perivascular space is the same as that of the CSF in all MRI sequences. The shape was linear when the image plane ran parallel to the blood vessels and round or oval when running perpendicular to the blood vessels, usually less than 3 mm in diameter. (v) Cerebral microbleeds: cerebral microbleeds are defined as the following changes in the images obtained with T2*-weighted gradient-echo sensitive to magnetizing effects, for instance, (1) small round or oval, clear boundary, homogeneity, lack of signal focus; (2) diameter in 2-5 mm, maximum 10 mm, and the lesion is surrounded by the brain parenchyma; (3) brain atrophy: reduced brain volume, but it was not associated with mainly specific focal lesions such as trauma and cerebral infarction.

The diagnostic criteria of vascular cognitive impairment (VCI) [37] indicate a continuum of clinical manifestations for cSVD patients. Mild VCI refers to impairment in at least one cognitive domain and mild to no impairment in instrumental activities of daily living (IADLs)/activities of daily living (ADLs) (independent of the motor/sensory sequelae of the vascular event). Major VCI refers to clinically significant deficits of sufficient severity in at least one cognitive domain (deficits may be present in multiple domains) and severe disruption to IADLs/ADLs (independent of the motor/sensory sequelae of the vascular event).

The inclusion criteria for the cSVD patients were as follows: (a) the patients aged in a range of 40–80 years old, (b) the patients or legal guardians agreed and signed informed consents, (c) the patient was confirmed to satisfy the diagnostic imaging criteria for cSVD and the diagnostic criteria for VCI, and (d) the patient was in mild to moderate cognitive impairment with a mini-mental state examination (MMSE) screening score in 9–24 points. Patients meeting all these criteria were included in our study. The exclusion criteria for the patients included: (a) the cognitive dysfunction caused by macrovascular and cardiogenic cerebral embolism confirmed by examination; (b) non-vascular causes of WM degeneration and pure AD; (c) those who have been confirmed to have brain tumors, brain trauma, cerebral parasitic diseases, encephalitis and other diseases that can cause cognitive impairment; (d) patients with severe speech, vision, hearing or mental disorders that affect cognitive testing and cognitive training; (e) patients suffered from depression and other neuropsychological disorders resulting in cognitive impairment; (f) patients have the history of alcohol and drug abuse; (g) patients combined with severe heart, liver, kidney endocrine system, and hematopoietic system diseases; and (h) participating in other clinical trials.

### 2.2. Assessments

Each patient was requested to attend the clinical assessments, such as a medical history inquiry, a neurological examination, and a series of neuropsychological tests, which included the verbal fluency test (VFT), digit span test (DST), and digit symbol substitution test (DSST).

The VFT is a widely used neuropsychological scale mainly to measure cognitive, verbal, and executive functions (Vaucheret Paz et al., 2020). It consists of three subtests to detect semantic, speech, and motion fluency, respectively. (1) semantic subtest: ask the participants to say as many animals, vegetable, or fruit words as possible in one minute; (2) speech subtest: ask the participants to say as many words as possible, starting with “Fa” in one minute; (3) motion subtest: participants were asked to say as many words as possible in one minute about an event that could occur in a particular location (e.g., kitchen).

The DST (Leung et al., 2011) consists of two parts, a digit forward and a digit backward. During the test process, the participants are asked to remember two numbers simultaneously, read by the researcher with the speed of one digit per second starting with the first set. If the participants pass the 2-digit number, then the 3-digit number is measured, and so on. If the participant does not pass the 8-digit number, the 8-digit number of the second set will be read, and when the participant passes the 8-digit number of the second set, the 9-digit number will be read. If the participant fails to pass the 8-digit number of the second set, his score will be “7-digit.”

The DSST was used to evaluate multiple aspects of cognitive function, such as executive function, processing speed, attention, and working memory (Baune et al., 2018). According to the diagram at the top of the scale, the participants filled in the matching numbers under each symbol in the table below as quickly as they could. Rating instructions are that the number of correct answers in 90 s is the final score, not including the numbers filled in during the practice. The number of correct answers will be counted as the total score.

### 2.3. Imaging data acquisition

All images were obtained on a GE 3T MRI scanner with an 8-channel phased-array head coil. The participant was requested to keep their eyes closed, relax but not fall asleep, and minimize head movement during the scanning. Functional images were collected with a gradient-echo echo-planar imaging (EPI) sequence with the following parameters: repetition time (TR) = 2,000 ms, echo time (TE) = 30 ms, flip angle (FA) = 90°, field of vision (FOV) = 240 mm × 240 mm, slice thickness = 3.5 mm, inter-slice gap = 0.7 mm, data matrix = 64 × 64, 33 interleaved axial slices coving the whole brain, and 240 volumes acquired in about 8 min. In addition, high-resolution brain structural images were acquired using a T1-weighed 3D BRAVO sequence with the following parameters: TR = 8.0 ms, TE = 3.0 ms, FA = 12°, data matrix = 256 × 256, FOV = 256 mm × 256 mm, slice thickness = 1 mm, and 188 sagittal slices covering the whole brain. The conventional T1-weighted and T2-weighted FLAIR images were acquired for clinical assessment. All MRI images for each participant were acquired in the same session.

### 2.4. Data pre-processing

The rs-fMRI data were preprocessed using the DPARSF toolbox^1^ based on Matlab2012a (Mathworks, Inc., Massachusetts). Before pre-processing the data, we visually inspected both brain functional and structural images and excluded the datasets with significant signal dropouts, distortion, and other quality problems. The procedure of pre-processing included: (1) removing the first 10 volumes to keep the magnetization equilibrium; (2) performing slice-timing and head-movement correction to remove effects caused by slice acquisition time differences and head movements; (3) conducting a linear co-registration between functional images and structural images for each participant; (4) regressed out signals of the WM and CSF, and head-movement parameters (Friston-24 model); (5) performed a non-linear transformation between structural images and template brain images of the Montreal Neurological Institute (MNI) space, and normalized functional images into the MNI space with 3 × 3 × 3 mm^3^ voxel size and smoothed with a Gaussian kernel of 5 mm full width at half maximum (FWHM), and (6) performed temporal band-pass filtering for the typical band (0.01–0.1 Hz), the slow-4 band (0.027–0.073 Hz), and the slow-5 band (0.01–0.027 Hz), respectively. In this study, the fMRI data for subjects with head motion displacement >2mm or rotation >2° in any axis (*x, y*, and *z*-axis) were discarded. In the calculation, we excluded the datasets for three participants because of their head-movement displacements exceeding 2 mm and the rotation exceeding 2°. A total of 41 cSVD patients and 32 HCs were finally included in the following analysis. There were also no significant group differences in the head motion between the two groups.

### 2.5. ALFF analysis

We first performed a voxel-wise Fast Fourier Transform (FFT) method for each participant to convert the filtered time series into the frequency domain to obtain the power spectrum. Since the power within a given frequency band is proportional to the square of the magnitude of that frequency component, we calculated the square root of the power spectrum within each frequency band and then averaged these square roots across three frequency bands: 0.01–0.1 Hz (typical band), 0.027–0.073 Hz (slow-4), and 0.01–0.027 Hz (slow-5) at each voxel. This averaged square root was taken as ALFF (Zang et al., 2007), which was assumed to reflect the absolute intensity of spontaneous brain activity.

### 2.6. Statistical analysis

#### 2.6.1. Demographic

A χ^2^-test was used to test between-group differences in sex. A *t*-test was used to test between-group differences in age. The Wilcoxon test was used to test the education level between groups. The statistical significance level was set at *p* < 0.05. Statistical analysis was conducted using SPSS (version 21.0).

#### 2.6.2. ALFF

The between-group difference in ALFF was conducted by PALM that is implemented in the DPARSF toolbox.^2^ In the calculations. A general linear model (GLM) was applied, and sex and age factors were regressed. A two-tailed non-parametric permutation test (5,000 times) was conducted to determine the differences between the two groups. For the multiple-comparison correction, we used cluster-forming threshold and family-wise error (FWE) methods. The significance level was set at *p* < 0.05 (cluster-forming threshold >2.3, voxel-wise FWE <0.05).

A mixed effect analysis was performed on the two groups and their ALFF on the slow-5 and slow-4 band, using a two-way repeated measures analysis of variance (ANOVA) to investigate the effects of group and frequency band with age, sex, and head motions (mean FD) as covariates. Group (cSVD and HCs) was used as a between-subjects factor and frequency band (slow-4 and slow-5) as a repeated measurements factor. In addition, *post hoc* tests were performed between slow-5 band and slow-4 bands on the cSVD patients within brain regions showing group and frequency band interactions (Gu et al., 2019; Wang Z. et al., 2020). In addition, we also took a threshold of 50 voxels to remove small clusters, which meant only a cluster size >50 voxels were reported. For the multiple-comparison correction, we used threshold free cluster enhancement (TFCE) and family-wise error (FWE) methods. The significance level was set at *p* < 0.01 (voxel-wise FWE < 0.01). The imaging results after the multiple-comparison correction were reported by the AAL atlas (Rolls et al., 2020).

To determine whether the ALFF value in each region varied with clinical measures, we performed the correlation analyses between the ALFF values for the significantly changed ALFF and each clinical variable (i.e., VFT, DSST, and DST). The threshold was set at *p*_two-tailed_ < 0.05.

## 3. Results

### 3.1. Demographic information

Table 1 lists the demographic information for both the cSVD and healthy controls, and clinical information for the cSVD group. No significant difference was found in sex between the two groups. The age and education level of the cSVD group were significantly higher than those of the healthy controls (*p < 0*.05).

### 3.2. ALFF in the typical frequency band (0.01–0.1 Hz)

**Figure 1:**
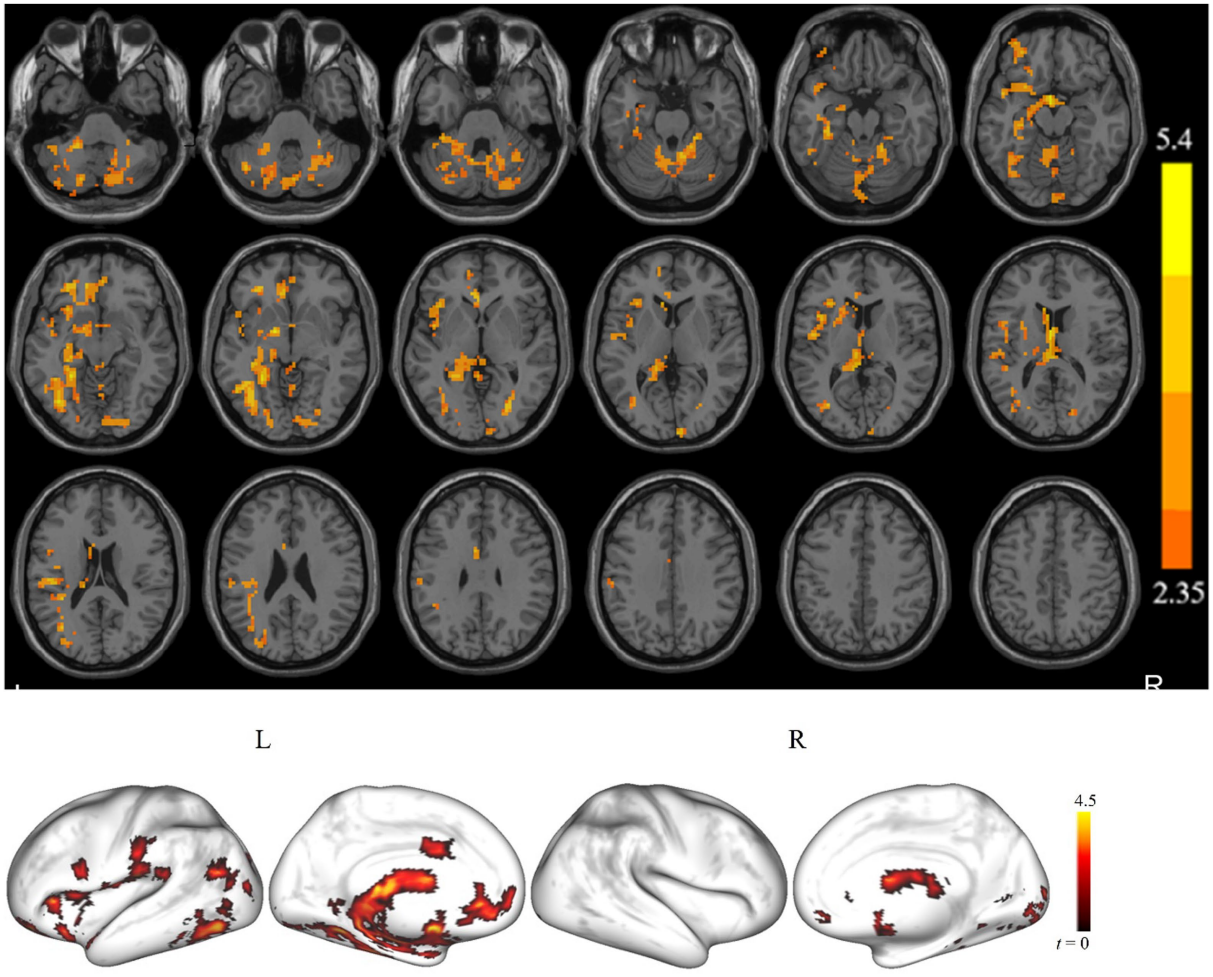
The brain regions show a significant difference in the amplitude of low-frequency fluctuation (ALFF) between the cSVD patients and the healthy controls in the typical frequency band (*p* < 0.05, cluster-forming threshold and FWE corrected). We found that the cSVD showed lower ALFF values in the one cluster in the left cerebellum, left hippocampus/parahippocampal, and left inferior occipital gyrus (IOG) of the controls.

Figure 1 displays clusters with significant between-group differences in ALFF. Compared with the controls, the cSVD patients showed significantly reduced ALFF in one cluster (cluster size = 3,019 voxels). The locations of the cluster are listed in Table 2. Specifically, the cSVD patients had significantly lower ALFF than the controls in the left fusiform gyrus/hippocampus/insula/inferior occipital gyrus/middle occipital gyrus/inferior frontal gyrus-medial orbital/rolandic operculum and the bilateral cerebellum.

**Table 2:** Brain areas showing significant differences in ALFF values under the typical (0.01–0.1 Hz) and slow-4 bands (0.027–0.073 Hz) between cSVD patients and HCs.

Frequency bands	Cluster size (Voxels)	MNI (*x y z*)	*t*	Brain regions
Typical	3,019	–6 30 –3	5.09	Cerebelum_8_R/L, Fusiform_L, Hippocampus_L, Cerebelum_Crus1/2_L, Insula_L, Occipital_Mid/Inf_L, Cerebelum_Crus1_R, Cerebelum_4_5_L, Cerebelum_6_L/R, Frontal_Inf_Orb_L, Rolandic_Oper_L
Slow-4	1,383	–3 0 –15	5.43	Cerebelum_crus1/2_L, Cerebelum_8_L, Hippocampus_L, Occipital_Mid_L, Fusiform_L, Cerebelum_crus1_L, Occipital_Inf_L

The significance threshold was set at *p* < 0.05, corrected for multiple comparisons using a cluster-forming threshold and the family-wise error (FWE) correction method. Coordinates of the peak voxel are shown in the Montreal Neurological Institute (MNI) space. The *t*-value corresponds to the peak voxel with a significant between-group difference in ALFF. The positive *t*-value represents a decrease (HCs > cSVD). Oper, operculum; Inf, inferior; Orb, orbital; Mid, middle; Sup, superior; L(R), left (right) hemisphere.

### 3.3. ALFF in slow-4 and slow-5

**Figure 2:**
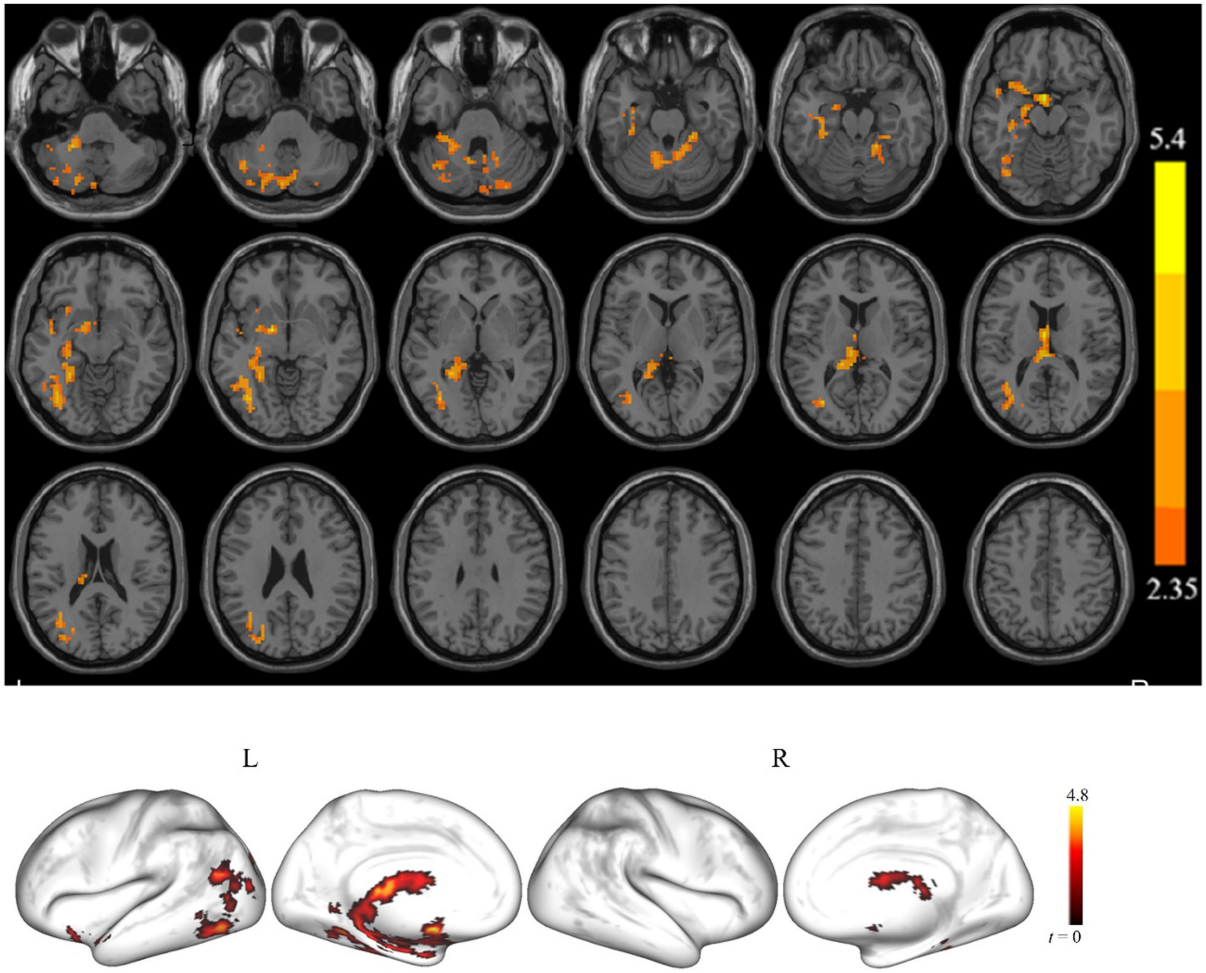
The brain regions showed a significant ALFF difference in the slow-4 band between the cSVD patients and the HCs (*p* < 0.05, cluster-forming threshold and FWE corrected). Hot (cold) colors represent higher (lower) ALFF in the HCs than in the cSVD patients.

Figure 2 shows the clusters with a significant difference in ALFF between the cSVD and controls for slow-4. For the slow-4, we found that the cSVD patients had significantly reduced ALFF in one cluster (voxel size = 1,383 voxels) in the left hemisphere, including the left cerebellum/hippocampus/fusiform gyrus/inferior occipital gyrus. As for the slow-5, no clusters showed a significant between-group difference in ALFF. The detailed information for the clusters is also listed in Table 2.

### 3.4. Interaction effect between group and frequency band

**Figure 3:**
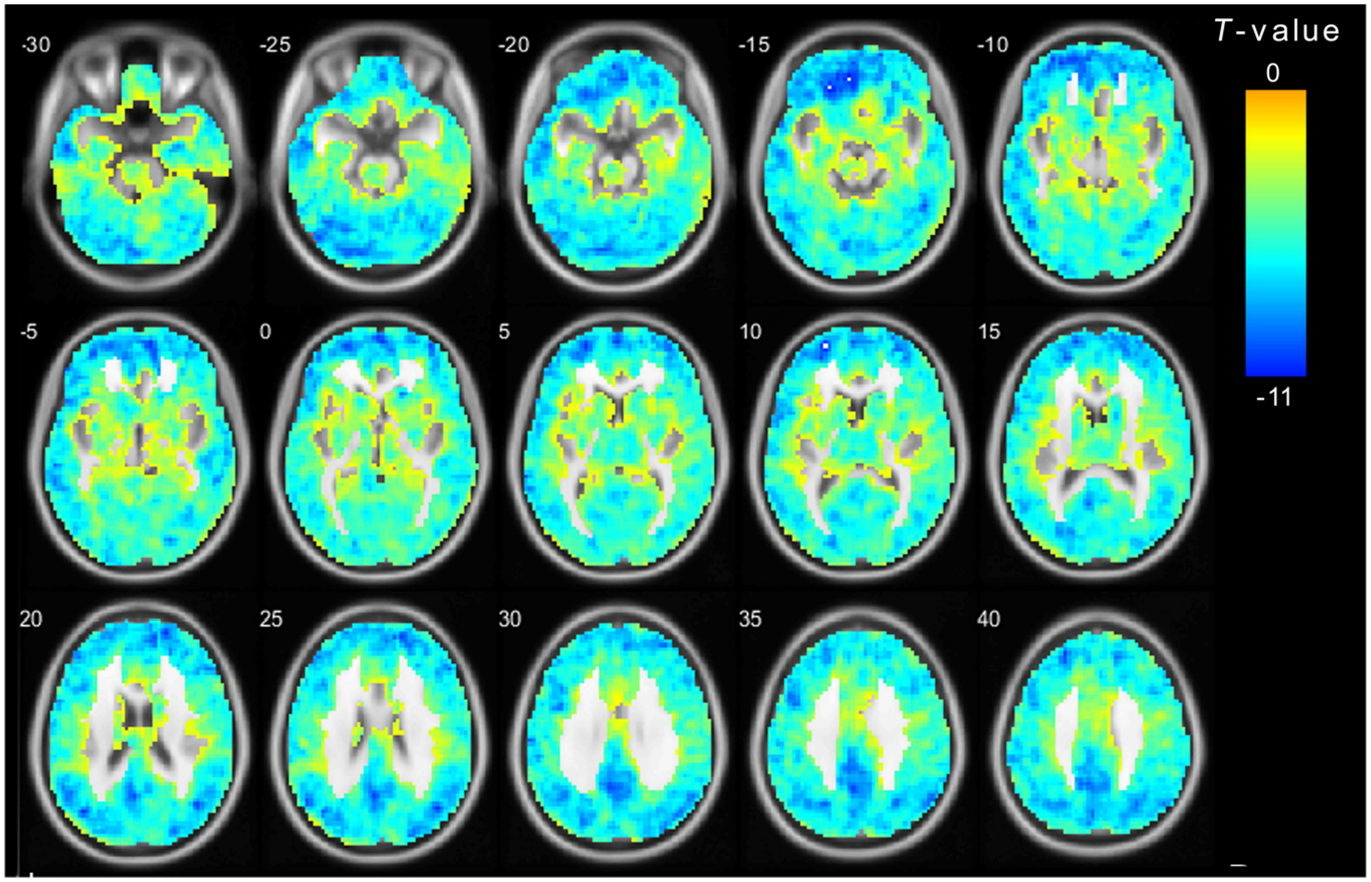
The main effect for frequency band on ALFF. The color bar on the right indicates the statistical *t*-value. Blue cold colors represent lower ALFF. The results were obtained by two-way repeated-measures ANOVA (TFCE and FWE corrected, voxel *p* < 0.01). TFCE, Threshold Free Cluster Enhancement.

**Figure 4:**
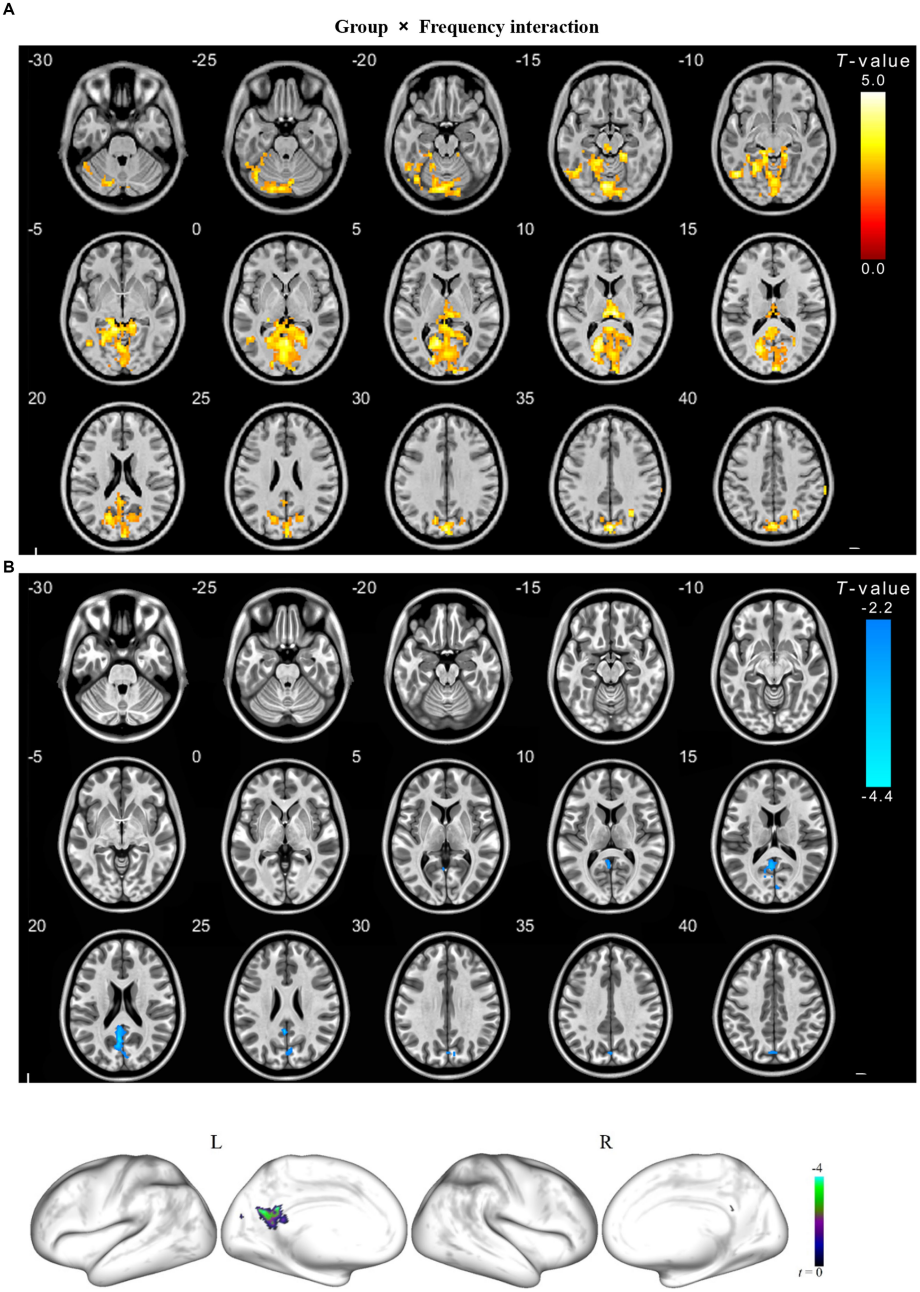
The interaction between frequency band and group on ALFF for cSVD patients. The results were obtained by a two-way repeated-measures ANOVA (*p* < 0.01, TFCE and FWE corrected) **(A)**; and then a post-hoc pair *t*-test shown in the **(B)** (*p* < 0.025, TFCE and FWE corrected), compared to the slow-5 band, cold overlays indicate lower ALFF in cSVD patients in the slow-4 band. TFCE, Threshold Free Cluster Enhancement.

We observed a significant interaction between the frequency band and group in the left calcarine, bilateral lingual, left cerebellum, bilateral precuneus, left cuneus, and right superior parietal gyrus (Table 3). Further *post hoc* tests showed significantly decreased ALFF values were identified in the left precuneus in the slow-4 band (Figures 3, 4).

**Table 3:** Significant interaction in ALFF between groups and frequency band.

Cluster	Brain regions	Cluster size (#voxels)	MNI (*x y z*)	*t* scores
A	Calcarine_L, Lingual_L, Lingual_R, Cerebelum_4_5_L, Cerebelum_Crus1_L, Cerebelum_6_L	769	–18 –72 12	4.83
B	Precuneus_L, Precuneus_R, Cuneus_L, Parietal_Sup_R	289	–3 –57 60	4.93

The interaction between frequency band and group on ALFF. The clusters were obtained by a two-way repeated-measures ANOVA (*p* < 0.01, TFCE and FWE corrected). Oper, operculum; Inf, inferior; Orb, orbital; Mid, middle; Sup, superior; L(R), left (right) hemisphere; TFCE, Threshold Free Cluster Enhancement.

#### 3.4.1. Relationship between ALFF and clinical performance

For each of the significant clusters, no significant correlation was found between ALFF values and any neuropsychological scales (*p* > 0.05).

## 4. Discussion

This study identified the specific alteration pattern of ALFF in cSVD patients under different frequency bands through a voxel-based whole-brain analysis. We found that the cSVD patients showed significantly lower ALFF in the cerebellum, hippocampus, and occipital cortex than the controls in the typical band and the slow-4 band. Additionally, *post hoc* analysis indicated that cSVD was associated with a wide range of abnormalities in brain activity with related frequency bands.

### 4.1. ALFF differences between cSVD and HC

We found that cSVD patients showed reduced ALFF in the cerebellum compared with the controls (Figure 1). Although previous studies had noted that the cerebellum is widely believed to be responsible for motor skills (Baune et al., 2018), coordination, and balance of visual-motor (Garfinkle et al., 2020), several studies (Koziol et al., 2014; Thomas et al., 2017; Seese, 2020) indicated that it is also involved in cognitive function and executive function. The decrease of microstructural integrity in the cerebellum of deep white matter hyperintensities patients was associated with dual-task gait speed. Dual-task gait speed was associated with three cognitive domains (global cognition, attention/processing speed, and executive function) (Ghanavati et al., 2018). Schaefer et al. (2014) applied rs-fMRI analysis methods (eigenvector centrality) in cSVD patients and showed reduced connectivity in frontoparietal networks, whereas connectivity increases in the cerebellum. Another important finding is a positive correlation between reaction time in the incongruent condition of the Stroop task with the eigenvector centralities of the cerebellar region. The functional connectome in cerebellar regions was increased while the functional connectome in frontoparietal cognitive networks was decreased; therefore, the author speculated that frontoparietal hypoconnectivity might be compensated by hyper-connectivity. For example, Li et al. (2018) found lower grey matter volume in the cerebellar in patients with early-onset AD than in healthy controls. Several studies (Li et al., 2013; Mascalchi et al., 2014) also found that MCI patients had reduced WM integrity. These studies of AD or MCI provided insight into MCI patients with brain structural or functional impairment in the cerebellum. Previous studies (Castellazzi et al., 2014) in MCI patients also reported that the FC of the cerebellum is lower than that in healthy controls and the cerebellar FC correlates positively with semantic fluency. Thus, we infer that the reduction of spontaneous activity in the cerebellum may be related to the cognitive dysfunction of cSVD patients.

This study also found aberrant spontaneous brain activity in the occipital cortex (Figure 1). In the pathological anatomy of cSVD, researchers found that cortical microinfarcts were presented more frequently in the parietal and occipital lobes, which were also related to the severity of cSVD (Ii et al., 2021). A comparison of our findings with those of other studies (De Marco et al., 2017; Marjańska et al., 2019) confirms dysfunction of the occipital lobe during AD and MCI. Gray matter volume of different brain regions was associated with cognitive ability in cSVD patients, and gray matter volume in the occipital thalamus was positively correlated with Montreal Cognitive Assessment scores (Wang Y. et al., 2020). Lambert et al. (2016) did longitudinal research into symptomatic cSVD to investigate. They showed that the rate of white matter hyperintensity progression is associated with increases in cortical gray matter atrophy rates in occipital lobes. Chen et al. (2020) found that the right inferior longitudinal fasciculus had a significantly negative correlation with total cognitive and episodic memory. It could be that the cSVD patients showed a relative decline of spontaneous brain activity in the occipital cortex, which might be part of the underlying brain function mechanism of cognitive dysfunction.

In addition, the current study also found abnormal spontaneous brain activity in the hippocampus of cSVD patients, suggesting that the occurrence of cognitive decline in cSVD may relate to the decreased activity in the hippocampus. The hippocampus is closely related to memory function and spatial navigational ability and plays a crucial role in all forms of declarative memory (including recognition) (Wixted and Squire, 2011). Jokinen et al. (2020) studied the relationship between grey matter volume and clinical symptoms of cSVD. They found a positive correlation between hippocampal volume and Vascular Dementia Assessment Scale–Cognitive Subscale, executive function, and memory. A study evaluated the influence of negative and positive cSVD-hippocampal subfield atrophy on episodic memory and frontal executive function (Wong et al., 2021). Hippocampal subfield atrophy worsened with increasing SVD severity, and atrophy in the subiculum, CA1, CA4, molecular layer, and dentate gyrus of the hippocampal were essential to poor episodic memory and frontal executive function. Xi et al. (2012) found that the low-frequency oscillation amplitudes of the right hippocampus, parahippocampal gyrus, left middle temporal gyrus and right ventromedial prefrontal cortex of MCI patients were reduced. The ALFF values of the hippocampus and parahippocampal gyrus had a positive correlation with the neuropsychological scale score. The hippocampal ALFF value of cSVD patients is abnormally decreased, and it is positively correlated with the MoCA score in our research. Many studies have affirmed the role of the hippocampus in the field of cognitive function, which may be a critical factor in the cognitive dysfunction of cSVD patients.

### 4.2. Group × frequency interaction

Different neurophysiological mechanisms have different physiological functions, including different oscillatory bands (Buzsáki and Draguhn, 2004). Previous studies had explored the differences in ALFF between brain areas in the slow-4 and slow-5 bands (Xiao et al., 2018; Wang Z. et al., 2020). Neural oscillations of different frequencies in the human brain may be sensitive to activities in different regions and can reflect different physiological functions of brain activities (Knyazev, 2007). Low-frequency oscillations are related to long-distance connections and the integration of large neuronal systems. In contrast, the high-frequency oscillations synchronization constitutes a functionally important neuronal peak time relationship in brain activity (Arnulfo et al., 2020). Connectivity between distal cortical regions is a valuable but expensive feature of cortical tissue and exists mainly between heterotypic (Oligschläger et al., 2017). It is generally believed that the slow-5 band is the main contributor of resting-state fluctuations in healthy humans (Zuo et al., 2010), and is more sensitive in the cortex (such as the temporal and Rolandic lobes). Our results are dissimilar to previous rs-fMRI studies in AD, showing more varied areas in the slow-5 band than the slow-4 band (Yang et al., 2020). Liu et al. (2014) also found that several temporal regions were more significant in the slow-5 band than the slow-4 band in AD patients. Our results are similar to previous studies in patients with cognitive impairment, which suggested that most cortical areas had more robust low-frequency oscillations and higher functional activities in the slow-5 band.

We observed a significant group × frequency interaction effect on the ALFF value in the left precuneus. These findings showed that the ALFF changes in CSVD patients with cognitive dysfunction are related to frequency bands, and the neurophysiological mechanism is unclear. Thus, we conclude that certain pathologic conditions in the precuneus may increase the influence of specific frequency bands. However, studies have found that precuneus plays a central role in many highly integrated tasks, including visual space imagery, situational memory extraction, and self-processing operations (Cavanna and Trimble, 2006). Hebscher et al. (2020) have also confirmed that the precuneus was closely related to human autobiographical memory. Luo et al. (2020) studied the functional connection between the precuneus and the cortical using resting-state fMRI and found that the human precuneus can be subdivided into six symmetrical and connected parcels. The precuneus has four sub-regions and acts as a mediator in the interaction of the default mode, multi-faceted attention, and frontoparietal control network. Precuneus and posterior cingulate cortex together belong to the posterior default mode network (DMN). The DMN is a specific neuronal circuitry to global brain function. Damage to the DMN often occurs in diseases such as Alzheimer’s disease, Parkinson’s disease, Epilepsy and attention deficit hyperactivity disorder (ADHD) (Mohan et al., 2016). A meta-analysis compared amnestic mild cognitive impairment patients (aMCI) with healthy control suggested that aMCI is associated with widespread aberrant regional spontaneous brain activity, predominantly involving the default mode, salience, and visual networks. In contrast, the increased severity of cognitive impairment in aMCI patients was associated with more significant decreases in ALFFs in the cuneus/precuneus cortices (Pan et al., 2017).

These findings indicated that frequency-specific ALFF could supplement important information for disease diagnosis as well as supply a new perspective for our understanding of the pathology of CSVD.

## 5. Limitations

This study has several limitations. First, the number of samples is not large enough, and age differences across cSVD patients or between patients and healthy control groups were not well addressed. Second, one of limitations of the present study is the 8-channel phased-array head coil employed for data acquisition, which represents the minimal number of channels. Using an advanced scanning sequence may need to be considered in future studies, which may potentially enhance qualities of scanned images. Third, we did not classify the sub-type of cSVD although there are many types of imaging performance and various pathophysiology of cSVD. Future study needs to recruit more patients and distinguish between different imaging findings to evaluate the degree of impairment of brain function in different types to guide the treatment. Fourth, the current study used the rs-fMRI only. It would be better to use multi-modal neuroimaging data, such as arterial spin labeling (ASL) and diffusion tensor imaging (DTI). The combination of multi-modal neuroimaging and neuropsychological scales might help us understand cognitive dysfunction progression in cSVD more thoroughly. Finally, the healthy controls included in our study were different in age from the CSVD group. Because age is a high-risk factor for the development of cerebral small vessel disease, we had great difficulty in finding age-matched healthy controls.

## 6. Conclusion

This study analyzed spontaneous brain activity in cSVD patients and showed abnormal ALFF in distinct brain regions in patients compared with controls. These abnormal brain regions were found in the cerebellum, occipital, and hippocampus in cSVD patients. These findings could reflect the pathogenesis of specific clinical manifestations of cSVD, especially cognitive decline.

## Data availability statement

The original contributions presented in the study are included in the article/supplementary materials, further inquiries can be directed to the corresponding authors.

## Ethics statement

The studies involving humans were approved by the Institutional Review Board of Zhongshan TCM Hospital (ClinicalTrials.gov identifier: 2016ZSZY-LLK-028). The studies were conducted in accordance with the local legislation and institutional requirements. The participants provided their written informed consent to participate in this study.

## Author contributions

SC: Data curation, Writing – original draft. RH: Methodology, Writing – original draft. MZ: Writing – original draft. XH: Writing – review & editing. SLing: Writing – review & editing. SLiu: Resources, Writing – review & editing. NY: Methodology, Writing – review & editing.

## Footnotes

^1^ http://rfmri.org/dpabi

^2^ http://rfmri.org/DPABI
